# Cognitive reappraisal training for parents of children with autism spectrum disorder

**DOI:** 10.3389/fpsyt.2022.995669

**Published:** 2022-10-17

**Authors:** Yael Enav, Antonio Y. Hardan, James J. Gross

**Affiliations:** ^1^Department of Counseling and Human Development, University of Haifa, Haifa, Israel; ^2^Department of Psychology, Stanford University, Stanford, CA, United States

**Keywords:** autism, parenting, cognitive reappraisal, reflective functioning, intervention

## Abstract

Parents of children with autism spectrum disorder (ASD) experience higher stress levels than those of typically developing children. The goal of the current study was to examine whether a mentalization-based intervention would enhance parental cognitive reappraisal, an adaptive form of emotion regulation associated with lower levels of stress. Findings from 27 parents who completed a short training indicated an improvement in cognitive reappraisal. In exploratory analyses, two different types of reappraisal were examined. The intervention-related improvement was found mainly with one type of reappraisal, namely reflective reappraisal that consist of cognitive reappraisal with mentalization characteristics. In light of the evidence indicating that high cognitive reappraisal and high reflective functioning are associated with quality caregiving, findings from the current study suggesting that a brief mentalization-based intervention supports ASD parents' cognitive reappraisal with mentalization characteristics are promising and warrant further investigation.

## Introduction

Parents of children with autism spectrum disorder (ASD) experience higher stress levels than parents of children with other neurodevelopmental disorders or children who are typically developing (1–5). Therefore, intervention programs may need to address parental stress, which in turn will benefit the child and the family. Effective emotion regulation (ER) strategies may reduce stress and other negative emotions and increase high-quality parent-child interactions (6, 7).

### Cognitive reappraisal

Because different forms of ER have different consequences, it is important to be specific about the types of ER that are (or are not) used in a particular family context. One particularly adaptive form of ER is cognitive reappraisal, a type of cognitive change ER (8, 9). Cognitive reappraisal has been found to change emotions in a sustained manner, and has been associated with lower levels of negative affect (10, 11). Successful cognitive reappraisal leads to better interpersonal functioning along with physical and psychological well-being (12, 13) and fewer mental health problems (14). In light of its positive effects, there has been a growing interest in the processes that support cognitive reappraisal.

### Mentalization and cognitive reappraisal

Mentalizing (also known as reflective functioning) refers to the creation of explanations of one's own or others' mental states, including thoughts, feelings, and intentions during an act of imagining and wondering (15). Mentalizing the experiences of self and others supports emotion regulation (16) and helps transform initial maladaptive thoughts into adaptive ones (15, 17). While mentalizing and wondering about others' states of mind, we imagine their perspective. Considering another person's point of view requires us to pay attention to and actively imagine that person's perspective (18), and this, in turn, promotes the understanding that each mind works differently and helps us become regulated and empathetic (19, 20). Mentalization may play a particularly central role in families of children with ASD, as it is hard to understand the children's thoughts and intentions, and often mentalization lacks a positive response from the child (21).

One reason mentalization may facilitate cognitive reappraisal is that reflective thinking initiates the development of complex representations and symbols (22, 23), leading to reappraisals with reflective characteristics (i.e., reflective appraisals) (24, 25). Reflective reappraisal is the process of reinterpretation of an event's meaning in order to down-regulate the experience of negative emotions that includes reflective characteristics (26, 27). In contrast, non-reflective reappraisals involve reinterpretations of an event's meaning that do not include reflective characteristics. For example: David, a 13-year-old, comes home from school angry and frustrated because he failed his math exam. When his dad asks for help setting the table for dinner, he shouts “Do it yourself” and slams his door. The first thought in his father's mind is: “He is so rude.” After focusing on his son's mind and imagining his thoughts, feelings, and intentions, a reappraisal might be: “I know how hard he studied. It must be so frustrating for him to fail the math exam. He is not a rude child.” In contrast, a non-reflective reappraisal could be: “He will calm down soon.”

The scientific literature has increasingly recognized the relationship between mentalization and cognitive reappraisal (24, 28, 29). For instance, Baylin (28) found mentalization initiated cognitive reappraisal. In a related vein, Fonagy et al. (24) argued mentalization plays a role in appraising an event in a way that promotes resilience. Moreover, in aversive situations, the automatic response is usually a negative thought, and the individual needs to reappraise it in a way that involves reflective, cognitive mentalizing (30). Similarly, Sharp et al. (29) presented a model for trauma intervention that integrated mentalization and cognitive reappraisal.

### The current study

This study is part of a larger study (31) that assessed the effect of mentalization based workshop on parental efficacy, parental belief in mailability of emotions and child's symptoms in parents of children with ASD. The goal of the current study was to examine whether and to what extent a mentalization-based intervention, bringing together aspects of psychodynamic and cognitive-behavioral focused on emotion regulation would affect parental cognitive reappraisal (the subjective interpretation made by the parent to an emotional stimuli) in parents of children with ASD. It was hypothesized that the intervention would lead to increased use of cognitive reappraisal, particularly reflective reappraisal.

## Method

### Participants

Parents of children with ASD were recruited to participate in a 4-week Reflective Parenting Workshop focused on emotion regulation (31). The present report is in an extension of a previous research, in the current investigation, the focus is on the effect of intervention on cognitive reappraisal.

Recruitment for the workshop occurred through distribution of fliers at clinics around the San Francisco Bay Area that provide services to families of children with autism. Twenty-seven intervention-group parents who completed an Emotion Interaction Questionnaire (EIQ) and ERQ (Emotion Regulation Questionnaire) before and after the workshop were included in the current study. Other inclusion criteria for the study included English proficiency, having a child with a diagnosis of autism spectrum disorder between the ages of 3 and 18 years, and completion of the workshop. This study was approved by the university's Institutional Review Board and registered in the Clinical Trials database. Table 1 presents participant demographics. As can be viewed from the table, all participating parents were married, and most were Caucasian women with two children. Over 80% of participants had academic degrees. One third were employed full-time, another one third were employed half-time, and the rest were either self-employed or homemakers. Approximately half of the children were between the ages 5 to 9, and half were between the ages 10 to 17. Most of the ASD children were high functioning.

**Table 1:** Frequency distribution on participants' demographic characteristics.

		***N* (%)**
Gender	Male	3 (11.1%)
	Female	24 (88.9%)
Marital status	Married	27 (100%)
Ethnicity	Caucasian	17 (63.0%)
	Pacific Islander	8 (29.6%)
	Other	2 (7.4%)
Number of children	Single child	3 (11.1%)
	Two children	19 (70.4%)
	Three children	4 (14.8%)
	Eight children	1 (3.7%)
Children's ages	5–9	14 (51.85)
	10–17	13 (48.15%)
Children's gender	Male	20 (74.07%)
	Female	7 (25.93%)
Children's functioning	Rating of 5	18 (66.67%)
	Rating of 4	2 (7.41%)
	Rating <= 3	6 (22.22%)
	Absent data	1 (3.70%)
Education	High school or GED	1 (3.7%)
	College or associate's degree	3 (11.1%)
	Bachelor's degree	6 (22.2%)
	Master's degree	13 (48.2%)
	PhD, MD, JD	3 (11.1%)
	Not mentioned	1 (3.7%)
Employment	Full-time	9 (33.3%)
	Part-time	9 (33.3%)
	Self-employed	2 (7.4%)
	Homemaker	6 (22.2%)

### Procedures

After signing consent forms, participants were screened for meeting the study inclusions criteria. Eligible participants then completed a 4-week face to face group workshop, one and a half hours once a week each session. Parenting workshop focused on reflective functioning and emotion regulation skills of parents of children with ASD based on the literature that associate high parental reflective functioning with high emotional regulation and both are related to high quality caregiving and parent-child relationship (32, 33). The intervention flow and key concepts: Session 1 - emotion, emotion regulation, different emotion regulation strategies with focus on cognitive reappraisal. Session 2 – The effect of parental emotion regulation on children, reflective functioning and holding the mind in mind, the challenges and opportunities in raising children with ASD. Session 3 – Participants are invited to share their emotional scenarios from the past week with the group. Role play of different parent-child emotional scenarios. Session 4 - Participants are invited to share their emotional scenarios from the past week with the group. Participants are invited to share anything about their participation in the workshop with an emphasis on next steps, what they took from the workshop, and anything that was particularly helpful. The participants filled out pre and post questionnaires. Participants were asked to complete emotion interaction questionnaire (EIQ) and an emotion regulation questionnaire (ERQ) in its trait version. The ERQ can be used in two different versions: one version is asking about using cognitive reappraisal during a specific time frame that can be considered as more situational. The other version is asking about using cognitive reappraisal generally, in life, without asking about a specific timeframe. Being non-related to a certain period and or situation, but to a general truth, this version can be considered as more trait related.

### Measures

For demographics, the following information was collected: age, gender, race, marital status, education status, and employment status. In addition, participants were asked to specify the number of children they have.

The Emotion Regulation Questionnaire (ERQ); (12) is self-report widely used measure of emotion regulation (34) and it was administered to assess parents' emotion regulation in trait format. It has good psychometric properties with above average internal consistency (Cronbach's α for reappraisal 0.75–0.82, and suppression 0.68–0.76, test–retest reliability across 3 months = 0.69), (12). The ERQ comprises 10 items divided into 2 subscales cognitive reappraisal and suppression. In this study we used the reappraisal subscale that consists of six items (e.g., “I control my emotions by changing the way I think about the situation I'm in”). Participants rated the degree to which they agreed with each statement on a 7-point scale (1 = strongly disagree, 7 = strongly agree).

The Emotion Interaction Questionnaire (EIQ, was developed by the research team) instructed parents first to describe a situation from the past week when they felt their negative emotions had an effect on their behavior, then to describe their reactions and thoughts during the situation and lastly to describe their thoughts after some time had passed.

### Data reduction

Two judges were trained to assess cognitive reappraisal, as well as its two subtypes, namely reflective reappraisal and non-reflective reappraisal. Each judge got a detailed explanation with the definition of each subtype, examples of situations that were assessed and coded by the research team and demonstrated each subtype and five situations that the judges were asked to assess and code as part of the training. Cognitive reappraisal was defined as reinterpreting the meaning of an emotional event or stimulus with the goal of influencing one's emotional response (35). Reflective reappraisal was scored as being present when the criteria for cognitive reappraisal were met, and the statement showed one of the following: (1). Awareness of the nature of mental states (22); (2). Recognition of developmental aspects of mental states (36); and (3). The effort to understand behavior based on mental states (37). An example that received a high score on reflective reappraisal is the following: “We had a doctor appointment for our son during school hours. I prepped him before school so it is not a surprise. He whined for an hour because he was missing recess.” After a time passed, “I was thinking how he felt, I gave him time for himself and I was happy that he loves school, I was thinking how change in routine is hard for him.” Non-reflective reappraisal was scored as being present when the criteria for cognitive reappraisal were met, but the statement did not meet the criteria for reflective reappraisal (there are no mentalization characteristics). Two judges got parental vignettes and had to assess on 1 to 7 Likert scale how much they saw evidence for cognitive reappraisal based on the vignette, where 1 = there is no evidence and 7 = there is strongly evidence. The judges were blind to the time point of assessment, i.e., whether the parents' description comes from the pre-or post assessment. The correlation between independent raters' scores was high for both reflective reappraisals (pre-intervention: *r* = 0.97, *p* < 0.001; post intervention: *r* = 0.93, *p* < 0.001) and non-reflective reappraisals (pre-intervention: *r* = 0.92, *p* < 0.001; post intervention: *r* = 0.91, *p* < 0.001). For this reason, the ratings were averaged across raters for each reappraisal type, at each time point (pre and post-intervention).

## Results

### Change in cognitive reappraisal pre- to post-intervention

To examine whether cognitive reappraisal scores, measured by the Emotion Regulation Questionnaire, increased with the intervention, we employed a paired samples *t*-test analysis. Results indicated that there was indeed such increase [pre-intervention cognitive reappraisal: M = 4.71, SE = 0.21; post-intervention cognitive reappraisal: M = 5.15, SE = 0.19; *t*_(26)_ = 2.24, *p* < 0.05, Cohen's D = 0.43].

**Figure 1:**
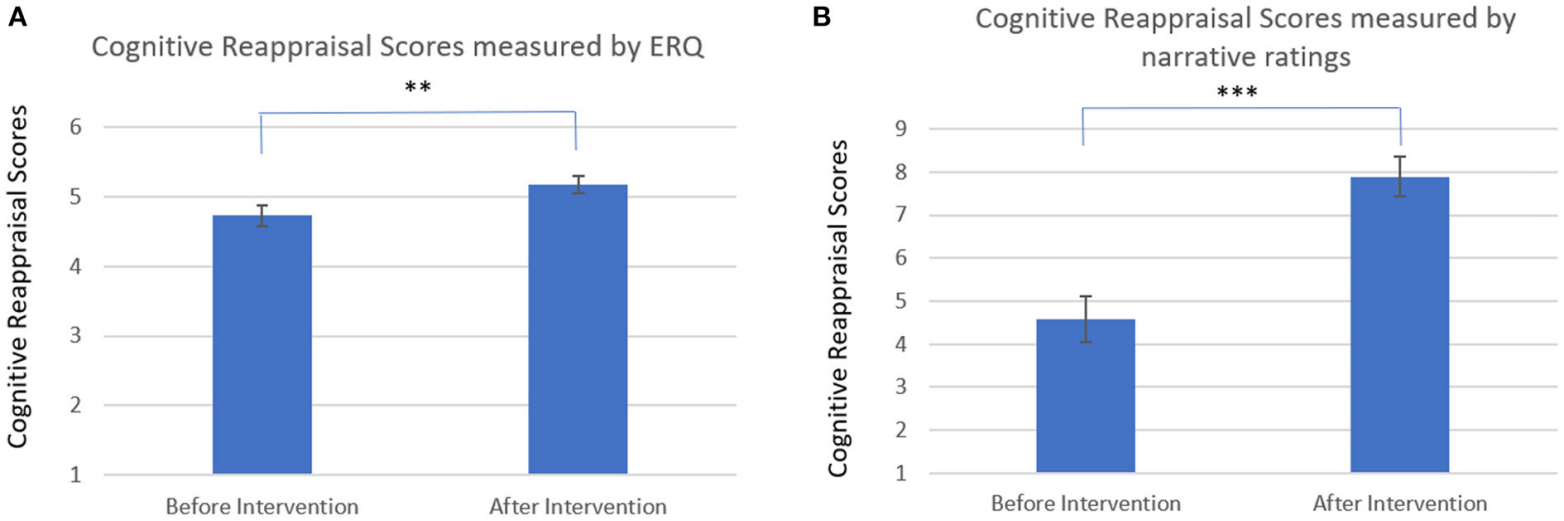
**(A)** Cognitive reappraisal scores measured by ERQ. Results indicate an increase in cognitive reappraisal, as measured by the emotion regulation questionnaire, after intervention. **(B)** Cognitive reappraisal scores measured by narrative ratings. Results indicate an increase in cognitive reappraisal, as measured by narrative ratings (situation-based cognitive reappraisal), after intervention. ***p < 0.001, **p < 0.01.

Next, we examined the increase in cognitive reappraisal scores as measured by narrative scoring. Cognitive reappraisal (CR) at each time point was computed as the sum of the two reappraisal types (CR = NR+RR). The increase in the computed CR measure was then examined using a paired samples *t*-test analysis. Results again indicated an increase between the two measurements [*t*_(26)_ = −4.46, *p* < 0.001, Cohen's D = 0.86; pre-intervention cognitive reappraisal: M = 4.57, SE = 0.53; post-intervention cognitive reappraisal: M = 7.89, SE = 0.46]. Figure 1A presents the results for the ERQ scoring, and Figure 1B presents the results for the narrative scoring. Interestingly, changes in the two indicators of cognitive reappraisal were not correlated (*r* = −0.17, ns), suggesting the difference between trait and situation-based assessments approaches.

### Change in reflective and non-reflective reappraisal pre- to post-intervention

**Figure 2:**
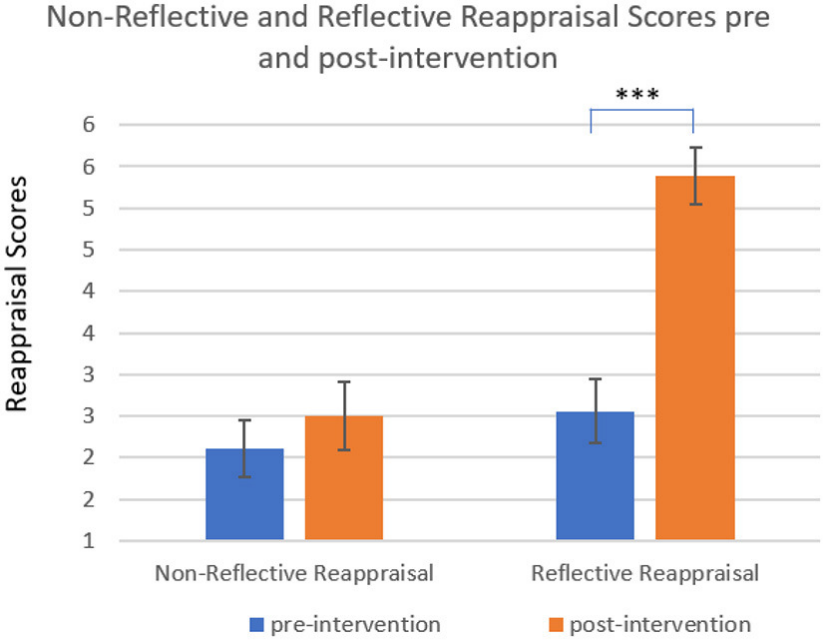
Non-reflective and reflective reappraisal scores, pre and post-intervention. Results indicate a significant difference between the two time points in reflective reappraisal scores. The difference in non-reflective reappraisal scores was non-significant. ***p < 0.001.

Following the primary analyses presented above, we proceeded to some broader, exploratory analyses, that examined change between pre and post-intervention in the two types of cognitive reappraisal, namely, reflective and non-reflective reappraisal (RR and NR, respectively). To this end, we employed a Repeated Measures ANOVA, with two within-subjects variables: time (pre and post) and reappraisal type (RR and NR). The analysis revealed three significant effects. The time effect was significant [*F*_(1,26)_ = 19.88, *p* < 0.001, $\eta_{\text{p}}^{2}$ = 0.43], so that pre-intervention scores (*M* = 2.29, *SE* = 0.27) were significantly lower than post-intervention scores (*M* = 3.94, *SE* = 0.23). The reappraisal type effect was also significant [*F*_(1,26)_ = 16.75, *p* < 0.001, $\eta_{\text{p}}^{2}$ = 0.39], so that NR scores (*M* = 2.26, *SE* = 0.24) were significantly lower than RR scores (*M* = 3.97, *SE* = 0.29). Finally, the time-reappraisal type interaction was significant, [*F*_(1,26)_ = 10.90, *p* < 0.01, $\eta_{\text{p}}^{2}$ = 0.30], so that in NR scores there wasn't a significant difference between the two time points (pre-intervention: *M* = 2.02, *SE* = 0.33; post-intervention: *M* = 2.50, *SE* = 0.41; n.s.), while in RR scores there was a significant difference between the two time points (pre-intervention: *M* = 2.56, *SE* = 0.38; post-intervention: *M* = 5.39, *SE* = 0.34). Figure 2 presents the time^*^reappraisal type interaction.

## Discussion

Our finding of an improvement in cognitive reappraisal after a mentalization-based intervention is in alignment with previous studies that demonstrate positive association between mentalization and successful emotion regulation (16). In particular, studies have shown that higher levels of mentalization are coupled with transformation from non-adaptive thoughts to balanced and adaptive ones (15, 17, 36) similar to the change we found in cognitive reappraisal. The possible improvement in emotion regulation after a short mentalization intervention is especially significant for parents of children with autism as they report higher levels of stress and prolong negative emotions compared to parents of typically developing children (2, 3, 5).

The increase in cognitive reappraisal is especially notable as it was evident using two different assessment methods. One is based on self-report that was given in a trait format. The second is based on a narrative scoring that was given in a state format. The improvement in cognitive reappraisal in a trait format after a short intervention is especially remarkable and might point to a possible underestimation of the true change. The improvement in state format includes ecological characteristics, based on daily situations. The fact that we did not see a correlation between the two methods may be due to the fact that correlations between trait and state measures are often weak (38, 39).

The increase in cognitive reappraisal post intervention was evidenced with reflective reappraisal but not with non-reflective reappraisal. One possible explanation for this specificity in effects could be related to the content of the intervention that is focused on mentalization. As mentalization facilitates reflective thinking, it makes sense that mentalization based intervention is leading to reappraisals with reflective characteristics (i.e., reflective reappraisals). This finding is consistent with previous studies that connect between mentalization and cognitive reappraisal with reflective characteristics. In particular studies present that the capacity to reflect on internal mental states of oneself and others is leading to cognitive appraisals that integrate qualities of mentalization (22, 24).

The improvement in reflective reappraisal following a mentalization-based intervention is important for parents of children with autism. They often have to work harder than parents of typically developing children to understand their children's behavior often in an absence of positive response from the child (21). As there is mounting evidence that high parental mentalization is associated with higher quality caregiving, attachment security, and successful emotion regulation (32, 33, 40) the possibility that short mentalization based intervention supports both parents' mentalization and emotion regulation is encouraging and support using both mentalization and emotion regulation principles in practice with parents of children with ASD.

While the current findings are presenting a potential positive impact of the mentalization-based workshop intervention for parents of children with ASD, this study has several limitations, which should be addressed in the future. First, the investigation does not have a control group, which limits our ability to determine whether change was related to the active intervention. Second, parents of children with ASD were included in the study but the diagnosis of ASD was not confirmed directly by study investigators. Instead, clinicians' reports were used to determine eligibility of parents. Third, the sample size of this study is relatively modest. Fourth, the measurement in this study were based on parents' reports. Finally, the children's ages ranged from 3 to 18, and we were underpowered to detect potential moderation by the children's age and sex.

## Author contributions

YE has lead the project and the paper. AH was a mentor and consultant from the Autism perspective. JG was a mentor and consultant from Emotion Regulation perspective. All authors contributed to the article and approved the submitted version.

## Conflict of interest

The authors declare that the research was conducted in the absence of any commercial or financial relationships that could be construed as a potential conflict of interest.

## Publisher's note

All claims expressed in this article are solely those of the authors and do not necessarily represent those of their affiliated organizations, or those of the publisher, the editors and the reviewers. Any product that may be evaluated in this article, or claim that may be made by its manufacturer, is not guaranteed or endorsed by the publisher.
